# How are different levels of social media engagement associated with mental distress and quality of life in young people living in deprived urban areas? A cross-sectional study in Bogotá, Buenos Aires and Lima

**DOI:** 10.1136/bmjopen-2025-102466

**Published:** 2025-09-17

**Authors:** Santiago Lucchetti, Luis I Brusco, Francisco Diez-Canseco, Carlos Gómez-Restrepo, Natividad Olivar, Sumiko Flores, Laura Montes Guzmán, Catherine Fung, Isabela Osorio Jaramillo, Heidy Sanchez, Diliniya Stanislaus Sureshkumar, Stefan Priebe

**Affiliations:** 1 Department of Psychiatry and Mental Health, University of Buenos Aires Faculty of Medicine, Buenos Aires, Argentina; 2 CRONICAS Centre of Excellence in Chronic Diseases, Universidad Peruana Cayetano Heredia, Lima, Peru; 3 Department of Clinical Epidemiology and Biostatistics, Pontificia Universidad Javeriana, Bogotá, Colombia; 4 Department of Psychiatry and Mental Health, Pontificia Universidad Javeriana, Bogota, Colombia; 5 Pontificia Universidad Javeriana Facultad de Medicina, Bogotá, Colombia; 6 Unit for Social and Community Psychiatry, Wolfson Institute of Population Health, Queen Mary University of London, London, UK; 7 University Medical Center Hamburg-Eppendorf Center for Psychosocial Medicine, Hamburg, Germany

**Keywords:** Social Media, Adolescents, Quality of Life, Depression & mood disorders, Anxiety disorders, Latin America

## Abstract

**Abstract:**

**Background:**

In deprived urban areas of South America, young people face heightened risks of mental disorders. Research suggests an association exists between social media engagement (SME), depression and anxiety.

**Objective:**

This study explored the associations of SME with symptoms of depression, anxiety and subjective quality of life among young people from South American deprived urban areas.

**Methods:**

Our cross-sectional survey study used an adapted version of the Multidimensional Facebook Intensity Scale to categorise 2399 participants into four SME groups: low, moderate, high and very high. Symptoms of depression (Patient Health Questionnaire-8), anxiety (Generalised Anxiety Disorder-7) and quality of life (Manchester Short Assessment) were assessed and compared using F and Tukey tests.

**Findings:**

Each step of increased SME was associated with more symptoms of depression and anxiety and poorer quality of life. Statistically significant differences were observed across all groups (p<0.001), and 15 out of 18 pairwise comparisons were statistically significant.

**Conclusions:**

The findings suggest an association exists between SME, increased mental distress and lower quality of life in young people from deprived South American urban areas. This influence seems to apply across the spectrum of engagement levels, not only to extremes. However, due to the cross-sectional nature of the study, causal relationships cannot be established.

**Implications:**

SME should be explored in clinical settings, as lower levels are associated with lower symptom levels and better quality of life. Policies addressing youth SME should be developed and evaluated in the challenging contexts of deprived urban areas.

**STRENGTHS AND LIMITATIONS OF THIS STUDY**

- The study successfully recruited a large sample from deprived urban areas in South American cities, despite challenges like participant access and researcher safety.
- Reliable, comparable findings were ensured through standardised methods and a low level of missing data.
- The observational design limits the ability to establish causality.
- Reliance on self-reported questionnaires may introduce response bias, and the non-representative sample may affect findings, though the associations remain strong.

## Introduction

### Background

 As the number of young people engaged in extensive use of smartphones and social media has risen in recent years, its link with mental health has received substantial attention. Part of the available literature highlights the negative effects of social media use, linking it to sleep deprivation and negative emotional impact.^1 2^ Previous studies have shown that smartphones and social media use are related to increases in mental distress, self-harm, body dissatisfaction and suicidality in young people.^1^,^4^ A meta-analysis conducted in 2022^5^ revealed a linear dose-response association between time spent on social media and depression. The association between social media use and quality of life has also been explored, showing how extensive social media use is related to reduced quality of life in young people.^6 7^

However, recent literature reviews propose different options to interpret the available evidence. Valkenburg *et al*^8^ state that, while most previous reviews suggest a weak association between more intensive social media use and poorer mental health, some defined this link as substantial. They suggest that this divergence may arise from the tendency to analyse results at an aggregate group level, potentially overlooking significant individual differences in how social media affects mental health. Moreover, the direction of these associations has been questioned. For instance, the link between poor sleep quality and social media use has been described as a vicious cycle^9^ or as a bidirectional link.^10^ Orben^11^ similarly highlights the unclear direction of the association between technology use and mental health.

Young people have a complex relationship with social media use. Although young people often perceive social media as a threat to their mental well-being or an ‘addiction’,^12^ social media also holds promise as a valuable tool for promoting positive mental health outcomes among young people. It offers opportunities for connection, communication, access to crucial mental health/medical information and peer interaction.^2 13^ Similarly, social media platforms have been used in innovative interventions to support youth mental health.^14^

A recent scoping review^15^ highlights that young people living in deprived urban areas of Latin America are exposed to multiple overlapping risk factors that significantly increase their vulnerability to mental health problems. Poverty, social exclusion, discrimination, violence and limited access to education, healthcare and basic services create sustained emotional stress and reduce opportunities for personal development. These challenges are compounded by family disruption, food insecurity and the lack of access to mental health services.

In these settings, young people may turn to social media for connection, information or support in the absence of other accessible resources, while simultaneously being exposed to risks such as misinformation, online harassment or social comparison. This potentially complex link stresses the need for further research on social media use and its implications for mental health. Henrich *et al*^16^ shed light on how most research in behavioural sciences is conducted on samples of western, educated, industrialised, rich and democratic societies. They warn about the risk of assuming that these samples are ‘standard’ and that the findings are representative of other populations. Regarding social media, Ghai *et al*^17^ make clear that most research on the topic focuses on populations from the Global North, and that further research is needed to determine potential differences with the Global South.

### Objective

Agreeing on the importance of developing a broader understanding of human behaviour, we endeavoured to explore social media use and mental health across three capital cities in South America, where research on this topic remains scarce.

We recruited young people from deprived urban areas, where access to resources for mental health promotion is often limited, and barriers to accessing mental health services may restrict young people from receiving help when needed. We explored their engagement with social media and its association with mental distress and perceived quality of life. In particular, we explored whether any association applies only to extreme groups or across the spectrum of different social media engagement (SME) levels in these settings.

## Methods

### Study design

We assessed young people within the research programme”Building resilience and resources to reduce depression and anxiety in young people from urban neighbourhoods in Latin America“ (OLA). This is a comprehensive multisite research programme aimed at investigating young people’s mental health and promoting resilience in three capital cities of South America: Bogotá (Colombia), Buenos Aires (Argentina) and Lima (Peru).^18^

The primary goal of this cross-sectional study was to identify which resources and activities influence symptoms of depression and anxiety in young people from deprived urban areas of Latin America. A core part was a comparison of resources and activities in young people with and without symptoms of depression and anxiety.

### Sample and inclusion criteria

Between April 2021 and November 2022, we recruited participants from the 50% most economically disadvantaged districts within each city. These were defined according to available data on deprivation levels. Bogotá and Lima used the United Nations Development Programme’s Human Development Index (HDI),^19^ while the Unsatisfied Basic Needs Index (NBI)^20^ was used in Buenos Aires. Both instruments determine vulnerability based on various domains of households, and the bottom 50% of districts in each city were considered eligible.

The recruitment and evaluation of participants partially coincided with the COVID-19 pandemic. Social restrictions were implemented in the early stages of the data collection period, which spanned 19 months. These restrictions, lasting approximately 10–15 months, fluctuated over time within individual cities and differed across cities.

Participants aged 15–16 or 20–24 were included in the adolescent and young adult groups respectively, using convenience sampling. The recruited participants were required to live in the eligible areas and to be able to provide informed consent. Additionally, adolescents needed to provide their assent and their parents/guardians’ informed consent. Consent forms were completed in person or online at their convenience, with the support of a researcher. Participants with known diagnoses of severe mental illness, cognitive impairment, who were illiterate or unable to provide consent, were not included.

Self-report questionnaires were employed to assess all outcomes of this study: the Patient Health Questionnaire-8 (PHQ-8)^21^ and Generalised Anxiety Disorder-7 (GAD-7)^22^ measured depression and anxiety symptoms respectively. Subjective quality of life was determined using the Manchester Short Assessment of Quality of Life (MANSA),^23^ and SME was assessed on an adapted version of the Multidimensional Facebook Intensity Scale (MFIS).^24 25^ A pilot study was conducted with 45 young participants from the same age range as the final sample, across the three participating cities. The aim was to assess the clarity, comprehensibility and cultural relevance of the questionnaire. Feedback was used to refine wording and layout. Responses from the pilot were not included in the final analysis.

As previously mentioned, this cross-sectional study is part of a larger prospective longitudinal research programme (OLA).^18^ Therefore, the sample size was calculated to detect predictors of recovery over a 1 year period. Assuming that a given predictor is present in 10% of the sample, a total of 762 participants would be required to detect a difference in recovery rates between 40% and 60%, with 90% power and a 5% significance level. To account for an expected 25% dropout rate, the baseline sample needed to include 1016 participants experiencing symptoms of depression and/or anxiety. Consequently, we aimed to recruit at least 340 participants with symptoms of depression and/or anxiety, and 340 controls in each of the three cities, for a total planned sample of 2040 participants.

To reduce bias, we recruited a varied sample of participants, primarily from schools and community centres, aiming to keep gender balance. Also, we used standardised assessment methods across sites. In preparation for the study, all researchers involved in data collection received training in all applied methods. Local stakeholders and community leaders were contacted to support the recruitment process.

The surveys were completed in person or online at participants’ convenience, with the support of a researcher. To ensure data integrity, a member of the research team reviewed each survey to ensure all items were completed appropriately. Participants were required to submit an identification document prior to completing informed consent. The research team used this information to prevent duplicate entries.

To ensure participant confidentiality, each individual was assigned a unique study ID number on recruitment. Documents linking personal identifiers to study IDs were accessible only to the research team and securely stored in locked cabinets at the universities or in password-protected digital files. A full description of recruitment and data collection procedures is outlined in the OLA protocol paper.^18^

### Patient and public involvement

In the early stages of this study, we established a Lived Experience Advisory Panel (LEAP) at each research site. The panel consisted of young people aged 15–24 who had experienced mental distress. The LEAP met two to three times annually to review the study’s progress and future plans. Their feedback helped refine recruitment and data collection processes.

We also partnered with arts organisations in the three research sites, which conducted activities with young people to explore their perspectives on mental health. Insights from these activities, along with focus groups involving adolescents, young adults and professionals, informed the development of the study’s data collection instruments.

Additionally, these arts organisations, alongside young people from their communities and LEAP members, are responsible for creating arts-based methods to disseminate the study’s results.

### Instruments and variables

#### Outcome variables

##### Patient Health Questionnaire-8 (PHQ-8)

This eight-item questionnaire assesses different symptoms of depression. Participants are asked to rate how often they have experienced each symptom over the past 2 weeks on a scale ranging from ‘0’ (not at all) to ‘3’ (nearly every day). It is widely used in clinical practice and research due to its brevity, simplicity and reliability in screening for depression.^21^

The PHQ-8 score represents the sum of scores for its eight items, measuring severity of depressive symptoms (total scores from 0 to 24).

##### Generalised Anxiety Disorder-7 (GAD-7)

It consists of seven questions that enquire about common symptoms of anxiety. Participants are asked to rate how often they have experienced each symptom over the past 2 weeks on a scale ranging from ‘0’ (not at all) to ‘3’ (nearly every day). It is widely used in both clinical and research settings due to its brevity, simplicity and reliability in screening for generalised anxiety disorder.^22^

The GAD-7 score represents the sum of scores for its seven items, measuring severity of generalised anxiety symptoms (total scores from 0 to 21).

##### Manchester Short Assessment of Quality of Life (MANSA)

This brief self-report questionnaire assesses an individual’s subjective quality of life across various domains: satisfaction with life as a whole, work/studies, finances, friendships, leisure activities, accommodation, personal safety, physical health, mental health, sexuality and family.

Participants rate their satisfaction on a scale ranging from 1 (not satisfied at all) to 7 (completely satisfied). This scale is widely used for clinical and research purposes and is a reliable instrument for assessing subjective quality of life.^23^

The MANSA score represents the mean score of the 12 items assessing satisfaction with different life domains on the scale, assessing perceived quality of life (mean scores from 1 to 7).

### Predictor variables

#### Social media engagement

We used an adapted version of the MFIS questionnaire, originally designed to assess Facebook use.^24 25^ The questionnaire was adapted for this study to investigate overall social media use, so the term ‘Facebook’ was replaced by ‘social media’. The first stage of our study, the Work Package 1 (WP1), was composed of 18 online focus groups and 12 online structured group conversations embedded into arts workshops.^26^ Adolescents, young adults and professionals who worked with these age groups took part in these activities, where youth mental health and resources were discussed. Although there was no separate validation study for the adapted version, these discussions and the reports from participants regarding how they used social media, informed the modifications to the MFIS. Four items were seen as redundant and removed, as they substantially overlapped or were almost identical (see items 2, 8, 10 and 12 of the original MFIS in online supplemental material SM1). Six items regarding social media interactions with others and seeking solutions through social media were incorporated (see items 10–15 of the adapted version in online supplemental material SM2).

As a result, a 15-item questionnaire was developed, with respondents rating statements on a 5-point Likert scale ranging from 1 (strongly disagree) to 5 (strongly agree). The SME score represents the sum of the responses to these 15 items, ranging from a minimum of 15 to a maximum of 75.

### Data analysis

We analysed this study’s data using the Statistical Package for the Social Sciences software, V.29.0.1.0, 2023. To examine the impact of SME on young people’s mental health and perceived quality of life, we categorised the sample of participants into four groups based on their level of SME. The low-engagement group comprised participants with scores ranging from 15 to 30, the moderate-engagement group from 31 to 45, the high-engagement group comprised participants with scores ranging from 46 to 60 and the very high-engagement group comprised participants with scores ranging from 61 to 75. The rationale behind this categorisation was that the low-engagement group represented answers on average between 1 (totally disagree) and 2 (disagree); the moderate-engagement group on average more than 2 and up to 3 (neither agree nor disagree) and the high-engagement group represented an average score for each item higher than 3 and up to 4 (agree). Finally, the very high-engagement group comprised participants who scored on average more than 4 and up to 5 (totally agree). This categorisation was chosen to align with the semantic structure of the response scale, rather than the score distribution in our sample. This ensures transparency and supports comparability in future studies. This provides clearly defined groups that can be replicated globally and ensures that differences between groups are transparent. Additionally, this approach allows for clinically meaningful interpretations that are accessible to all readers without requiring the translation of statistical coefficients into effect sizes or other abstract metrics.

The risk of depression, anxiety and the level of subjective quality of life were compared across the four groups using an F test followed by post hoc tests. Specifically, the Tukey honestly significant difference and Student–Newman–Keuls tests were used. Pearson correlation analysis was employed to examine the relationships among variables, with a significance threshold set at p<0.05.

To complement the analyses of categorical data, we further analysed the data treating all variables as continuous. A path analysis was then conducted using structural equation modelling, with SME as the independent variable and depression, anxiety and quality of life as the dependent variables.

## Findings

**Figure 1:**
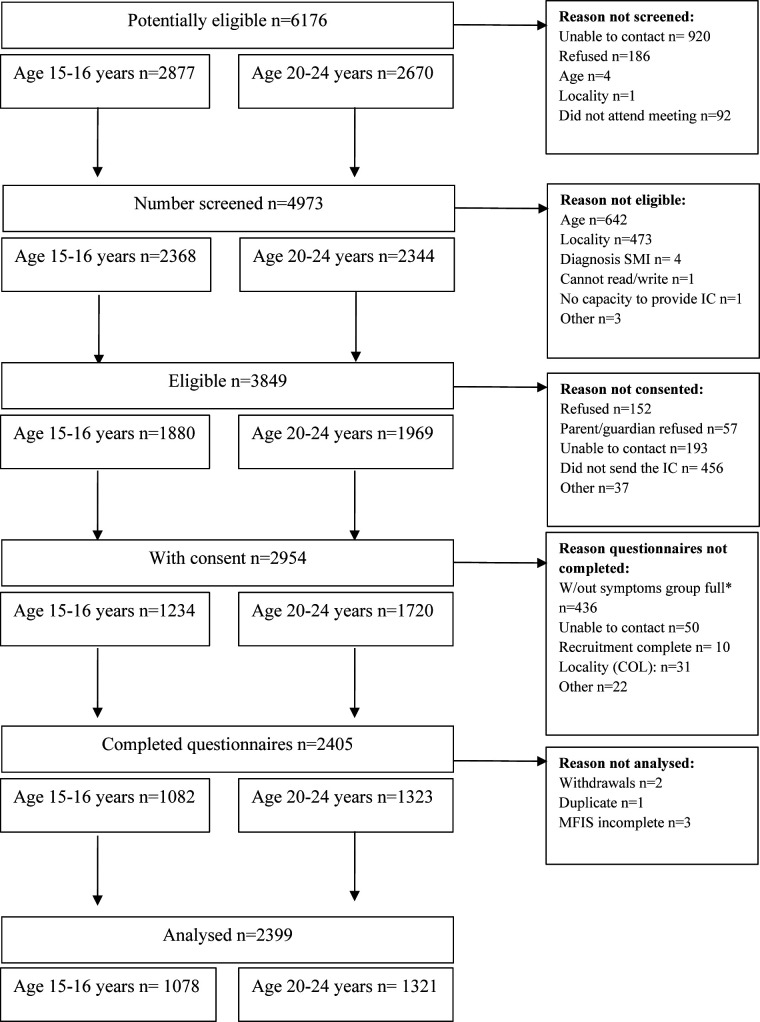
Flow chart showing numbers of young people at each stage of recruitment, data collection and analysis. *When the number of participants without symptoms aimed for in the protocol was reached, only participants with symptoms were included in the study. MFIS, Multidimensional Facebook Intensity Scale; SMI, severe mental illness; IC, informed consent; COL, Colombia.

After screening 4973 potential participants, 2954 provided consent and 2405 completed the questionnaires. Due to withdrawals, duplicated participants and incomplete answers to the MFIS questionnaires, six participants were excluded before the analyses (see figure 1, adapted from Gómez-Restrepo *et al*^27^). The sociodemographic characteristics of the total sample are presented in table 1.

**Table 1:** Sociodemographic characteristics of the total sample

Sample variables	N (%)	Total (N)
Gender		
Male: n (%)	813 (34%)	2399
Female: n (%)	1559 (65%)	
Other gender: n (%)	27 (1%)	
Age group		
Adolescents (15–16 years): n (%)	1078 (45%)	2399
Young adults (20–24 years): n (%)	1321 (55%)	
City		
Bogotá: n (%)	963 (40%)	2399
Buenos Aires: n (%)	621 (26%)	
Lima: n (%)	815 (34%)	

Out of the total sample of 2399 individuals, 342 (14%) presented low SME, 1202 (50%) moderate engagement, 773 (32%) high engagement and 82 (4%) very high engagement. Sociodemographic characteristics stratified by SME group are shown in table 2.

**Table 2:** Sociodemographic characteristics for each social media engagement (SME) group

Variable	Total sample (n=2399)	Low SME (n=342)	Moderate SME (n=1202)	High SME (n=773)	Very high SME (n=82)
Adolescents	1078 (44.9%)	146 (42.7%)	530 (44.1%)	368 (47.6%)	34 (41.5%)
Young adults	1321 (55.1%)	196 (57.3%)	672 (55.9%)	405 (52.4%)	48 (58.5%)
Male	813 (33.9%)	128 (37.4%)	406 (33.8%)	259 (33.5%)	20 (24.4%)
Female	1559 (64.9%)	213 (62.3%)	780 (64.9%)	505 (65.3%)	61 (74.4%)
Other gender	27 (1.1%)	1 (0.3%)	16 (1.3%)	9 (1.2%)	1 (1.2%)

A χ^2^ test was conducted to assess differences in age and gender distribution across SME groups. The results indicated no significant association for age group (p=0.30) or gender (p=0.27).

Among the 2399 participants, the response rate for the PHQ-8, GAD-7 and MFIS was 100%. The MANSA was completed by 2347 participants, yielding a response rate of 97.8%. According to previous research,^21 22^ mean PHQ-8 and GAD-7 scores for the total sample correspond to moderate depression and mild anxiety. Table 3 shows the symptoms of depression, anxiety and the quality-of-life mean scores for the total sample and the four groups of SME.

**Table 3:** Mean scores of depression, anxiety and quality of life of the total sample and by social media engagement (SME) group

	Mean scores					F score†	Sig
	Total sample (n=2399)*	Low engagement (n=342)	Moderate engagement (n=1202)	High engagement (n=773)	Very high engagement (n=82)		
Depression	10.01 (SD=5.98)	8.23 (SD=6.3)	9.62 (SD=5.9)	11.08 (SD=5.7)	13.07 (SD=5.3)	28.232	<0.001
Anxiety	8.51 (SD=4.92)	7.53 (SD=5.4)	8.15 (SD=4.8)	9.22 (SD=4.7)	11.28 (SD=4.6)	21.091	<0.001
Quality of life	4.65 (SD=1.08)	4.77 (SD=1.2)	4.71 (SD=1.0)	4.54 (SD=1.0)	4.29 (SD=1.3)	8.476	<0.001

* Total sample for QoL (Manchester Short Assessment (MANSA) questionnaire) (n=2347).

† For the complete results of the ANOVA test, see online supplemental table 5

Out of the 2399 participants, 52 did not complete the MANSA questionnaire and were therefore excluded from the QoL and SME analyses. Consequently, QoL and SME analyses were conducted on this subset of 2347 participants.

Each group exhibited a progressively higher prevalence of symptoms and a lower subjective quality of life than those with lower engagement with social media. These differences across the four groups were statistically significant for each of the outcome measures.

Pairwise comparisons among the four SME groups were conducted to identify specific statistically significant differences in depression and anxiety symptoms, as well as subjective quality of life. The results of these analyses are shown in table 4.

**Table 4:** Pairwise comparisons between social media engagement (SME) groups according to Patient Health Questionnaire-8 (PHQ-8), Generalised Anxiety Disorder-7 (GAD-7) and Manchester Short Assessment (MANSA) scores

Pairwise comparisons					
Measure	Engagement group	Low engagement	Moderate engagement	High engagement	Very high engagement
		Difference of means			
Depression*	Low engagement	–	−1.392†	−2.853†	−4.845†
	Moderate engagement	1.392†	–	−1.462†	−3.453†
	High engagement	2.853†	1.462†	–	−1.992†
	Very high engagement	4.845†	3.453†	1.992†	–
Anxiety‡	Low engagement	–	−0.625	−1.687†	−3.751†
	Moderate engagement	0.625	–	−1.062†	−3.127†
	High engagement	1.687†	1.062†	–	−2.064†
	Very high engagement	3.751†	3.127†	2.064†	–
Quality of life§	Low engagement	–	0.058	0.232†	0.480†
	Moderate engagement	−0.058	–	0.174†	0.422†
	High engagement	−0.232†	−0.174†	–	0.248
	Very high engagement	−0.480†	−0.422†	−0.248	–

* For details on the pairwise comparisons, see online supplemental table 6.

† The difference in means is significant at the 0.05 level.

‡ For details on the pairwise comparisons, see online supplemental table 7.

§ For details on the pairwise comparisons, see online supplemental table 8.

Among the 18 pairwise comparisons between single groups tested, 15 presented statistically significant differences. The comparisons between groups with low and moderate engagement on the GAD-7 and MANSA, and between groups with high and very high engagement on the MANSA failed to reach statistical significance.

The path analysis conducted showed that SME has a statistically significant effect on depression and anxiety, both in a positive direction, and on quality of life, in a negative direction. SME accounts for between 1% and 4% of the variance in these outcomes. Full details, including descriptive statistics and model results, are provided in online supplemental tables 9–11.

Descriptive analyses by gender and age group showed similar trends across subgroups, with higher SME generally associated with higher levels of depression and anxiety symptoms and lower quality of life. Among young adults, moderate SME was linked to slightly better quality of life than low SME, but overall patterns remained consistent. Full subgroup scores are provided in online supplemental tables 12 and 13.

## Discussion

The findings suggest that higher SME is associated with higher depression and anxiety symptom levels and poorer quality of life in young people living in deprived areas of large cities in South America. The highest SME group has substantially higher symptom levels of depression (8.23 vs 13.07) and anxiety (7.53 vs 11.28) than the lowest group.

However, higher symptom levels and lower quality of life were not restricted to extremely high SME. Each group with higher SME had more symptoms of depression and anxiety and poorer quality of life. This association applies across the full spectrum of engagement, although the size of the differences varies for each step of higher SME.

### Strengths and limitations

The strengths of our study include recruiting a large sample in the challenging context of deprived urban areas in large South American cities, where access to participants and guaranteeing the safety of researchers can be difficult. As such, it is the first large study on the link between SME and mental health in these settings. We used standardised methods to ensure the reliability and comparability of our findings, and the low level of missing data strengthens the analysis.

However, the study has some limitations. First, our cross-sectional design does not allow for causal inferences. While our findings show an association between higher SME and greater psychological distress, we cannot determine the direction of this relationship. It is possible that some participants increased their social media use as a coping strategy in response to pre-existing depression or anxiety symptoms, rather than SME contributing to their distress. Second, reliance solely on self-report questionnaires introduces the potential for response bias and measurement error, which may influence the accuracy and generalisability of our findings. However, the instruments used in this study have been widely tested in research and clinical practice internationally. Regarding the instruments, we assume face validity for the adapted MFIS scale, but it was not specifically validated in deprived South American areas. Third, the sample is not representative, which is likely to affect the identified levels of distress and SME more than their associations, which are more robust against selection biases. Additionally, the findings reflect results on group levels and do not exclude that SMEs may have a positive effect in individual cases.

Finally, data collection took place during the COVID-19 pandemic, which may have influenced both social media use and mental health outcomes. However, pandemic-related restrictions in South American cities varied considerably across time and location, with differences in intensity, enforcement and duration. As our study was not designed to systematically capture these contextual variations, we were unable to determine their potential effects on the observed associations.

### Comparison with previous literature

Prior studies link heightened SME to adverse mental health outcomes, including mood and anxiety disorders, sleep disturbances, mental distress, self-harm, body dissatisfaction, suicidality, cyberbullying and internet addiction.^1^,^4^

Our findings are consistent with this narrative, showing that higher SME is associated with an elevated risk of mental distress and a lower QoL. A multivariable analysis on the same sample^27^ confirmed the link between high SME and depression and anxiety symptoms. Moreover, our findings suggest a noteworthy gradual trend: increasing SME corresponds with escalating levels of mental distress and declining quality of life across the spectrum.

Existing literature has also explored the potential positive aspects of social media use.^2 14 15 26 28^ These studies argue that social media platforms may promote mental health, depending on how they are used (eg, for information dissemination, peer support, communication and interaction with experts).

Our findings align with those published by Liu *et al*,^5^ which show a linear relationship between depression and time spent on social media. Both studies suggest that an increase in SME is associated with more symptoms. However, as Valkenburg *et al*^8^ highlighted, this applies at the group level and does not exclude the possibility that some individuals might benefit from high SME.

Furthermore, while our data show an association, we cannot determine whether SME contributes to poorer mental health, or whether young people already experiencing psychological distress are more likely to engage with social media. This highlights the importance of considering both directions of influence when interpreting group-level trends. This possibility has been noted in recent longitudinal and meta-analytic studies,^10^,^12^ and reinforces the need for caution when interpreting the results of a cross-sectional study.

Our sample was entirely recruited from economically disadvantaged and understudied urban areas in South America. Evidence on the impact of social media and on youth mental health is scarce in the Global South. Furthermore, the available literature is inconclusive, suggesting both positive and negative associations between SME and well-being.^17^ Our findings align with studies from other parts of the world despite differing contexts. However, further research is needed to explore the effects of SME on more diverse populations and better understand its complex relationship with mental health.

Structural and environmental conditions specific to deprived urban areas may influence both social media use and mental health. Factors such as limited access to safe recreational spaces, economic vulnerability, family instability and lack of access to health services^15^ may influence how young people engage with social media or the risk of developing mental disorders. These contextual factors could affect the associations observed and these may differ from those in populations typically studied in high-income countries. Future research should explore how such conditions shape the role of social media in mental health, particularly in underserved settings like those included in our study.

It is also important to note that data collection took place during the COVID-19 pandemic, a period marked by increased reliance on digital platforms and heightened psychological vulnerability among young people. These contextual factors may have influenced both SME and mental health outcomes. However, pandemic-related restrictions in South American cities varied substantially across time and location, with considerable differences in intensity, enforcement and duration. As our study was not designed to systematically capture these variations, we were unable to assess their potential moderating effects on the observed associations.

### Implications

#### Clinical relevance

While this study does not establish a causal relationship, the associations between SME and symptoms of depression and anxiety and quality of life, suggest that SME may be a relevant factor to consider in clinical assessments of youth mental health.

Given the link between SME and mental distress, clinicians should explore not only the amount of time young patients spend on social media but also their patterns of use, emotional responses and motivations. These qualitative aspects may help to understand as to whether SME contributes to, exacerbates, or reflects underlying mental health difficulties.

Interventions aimed at supporting young people experiencing mental distress may include helping them develop a more intentional and reflective approach to SME. Rather than focusing solely on reducing screen time, clinicians might encourage young people to evaluate how specific online interactions affect their mood and well-being. Young people may be encouraged to identify alternative activities of interest that are accessible within their social and economic contexts. The findings of this study suggest that young people across the spectrum of SME, and not only those with extremely high levels of SME, may benefit from such interventions.

#### Future research

Further research is needed to explore the exact processes linking social media use with mental distress. Research should consider the nuanced interplay between SME patterns, qualitative aspects of their use and their effects on mental health and QoL. Our study focuses on the quantitative aspect of SME. Other qualitative studies^26 28^ have explored specific negative, and in individual cases also positive, aspects of SME.

Longitudinal studies would provide information on how the association between mental health and SME patterns evolves over time. Experimental studies may test the causality in the association and explore how specific characteristics of SME, such as content type or interaction with others, can influence mental health and QoL.

Moreover, future research should aim to address gaps in knowledge about SME and mental health in populations from the Global South. A deeper understanding of its effects across diverse contexts is essential for generating more reliable and globally relevant insights.

#### Policy implications

Social media’s impact on youth has sparked global attention, with legal restrictions and the removal of addictive features debated in the USA, UK and EU.^29^,^31^ Social media apps are designed to engage users and encourage them to spend time on their screens without noticing. This makes it difficult, especially for young people, to reduce screen time. However, a total ban on social media apps for young people also seems complex to implement and may not be ideal in every case. Reliable data is needed to inform these debates and guide the general public, potentially reshaping youth’s relationship with social media.

Our study focused on young people living in deprived urban areas of South America. Information campaigns alone may not be enough to reshape social media use where alternative activities are limited, financial resources scarce, and in-person socialising or public spaces may be often unsafe. It is necessary to develop and test interventions that are effective yet realistic for each context.

### Conclusions

This study found that higher SME is associated with increased symptoms of depression and anxiety, as well as lower subjective quality of life, among young people living in deprived urban areas of South America. These associations were observed across the full spectrum of SME, with higher engagement levels corresponding to greater reported distress.

Our findings contribute to the limited body of research on SME and mental health in the Global South, aligning with studies conducted in higher-income countries. However, the current evidence remains inconclusive regarding the direction of these associations. Given the cross-sectional design of this study, we cannot determine whether SME contributes to mental health difficulties, whether pre-existing distress leads to increased SME, or whether a bidirectional relationship exists.

Further research, particularly longitudinal and experimental studies, is necessary to clarify the mechanisms underlying these associations and to explore the role of specific patterns of social media use. Investigating both risks and potential benefits of SME in diverse social and economic contexts will be critical for developing evidence-based recommendations.

While public health and clinical discussions often emphasise the need to reduce social media use, strategies should move beyond a simplistic focus on screen time. Different types of social media use may have varying impacts, and these effects may also differ among individuals with diverse characteristics. Moreover, future interventions should consider the broader social environment and explore accessible, meaningful alternatives to SME for young people in disadvantaged settings. Collaboration with community organisations and local stakeholders may enhance the feasibility and impact of such efforts.

## Supplementary material

10.1136/bmjopen-2025-102466 online supplemental material 1

## Data Availability

Data are available upon reasonable request.

## References

[1] Abi-Jaoude E, Naylor KT, Pignatiello A (2020). Smartphones, social media use and youth mental health. CMAJ.

[2] Gupta C, Jogdand DS, Kumar M (2022). Reviewing the Impact of Social Media on the Mental Health of Adolescents and Young Adults. Cureus.

[3] Lin LY, Sidani JE, Shensa A (2016). Association between Social Media Use and Depression among U.S. Young Adults. Depress Anxiety.

[4] Shannon H, Bush K, Villeneuve PJ (2022). Problematic Social Media Use in Adolescents and Young Adults: Systematic Review and Meta-analysis. JMIR Ment Health.

[5] Liu M, Kamper-DeMarco KE, Zhang J (2022). Time Spent on Social Media and Risk of Depression in Adolescents: A Dose–Response Meta-Analysis. IJERPH.

[6] Aliverdi F, Farajidana H, Tourzani ZM (2022). Social networks and internet emotional relationships on mental health and quality of life in students: structural equation modelling. BMC Psychiatry.

[7] Karacic S, Oreskovic S (2017). Internet Addiction and Mental Health Status of Adolescents in Croatia and Germany. Psychiatr Danub.

[8] Valkenburg PM, Meier A, Beyens I (2022). Social media use and its impact on adolescent mental health: An umbrella review of the evidence. Curr Opin Psychol.

[9] Pagano M, Bacaro V, Crocetti E (2023). “Using digital media or sleeping … that is the question”. A meta-analysis on digital media use and unhealthy sleep in adolescence. Comput Human Behav.

[10] Bauducco S, Pillion M, Bartel K (2024). A bidirectional model of sleep and technology use: A theoretical review of How much, for whom, and which mechanisms. Sleep Med Rev.

[11] Orben A (2020). Teenagers, screens and social media: a narrative review of reviews and key studies. Soc Psychiatry Psychiatr Epidemiol.

[12] O’Reilly M, Dogra N, Whiteman N (2018). Is social media bad for mental health and wellbeing? Exploring the perspectives of adolescents. Clin Child Psychol Psychiatry.

[13] O’Reilly M, Dogra N, Hughes J (2019). Potential of social media in promoting mental health in adolescents. Health Promot Int.

[14] Ridout B, Campbell A (2018). The Use of Social Networking Sites in Mental Health Interventions for Young People: Systematic Review. J Med Internet Res.

[15] Sánchez-Castro JC, Pilz González L, Arias-Murcia SE (2024). Mental health among adolescents exposed to social inequality in Latin America and the Caribbean: a scoping review. Front Public Health.

[16] Henrich J, Heine SJ, Norenzayan A (2010). The weirdest people in the world?. Behav Brain Sci.

[17] Ghai S, Magis-Weinberg L, Stoilova M (2022). Social media and adolescent well-being in the Global South. Curr Opin Psychol.

[18] Priebe S, Fung C, Brusco LI (2021). Which resources help young people to prevent and overcome mental distress in deprived urban areas in Latin America? A protocol for a prospective cohort study. BMJ Open.

[19] (2024). Undp.org. https://hdr.undp.org/data-center/human-development-index#/indicies/HDI.

[20] (2024). WebINDEC - Sociedad / Condiciones de vida / Necesidades básicas insatisfechas. Gob.ar.

[21] Kroenke K, Strine TW, Spitzer RL (2009). The PHQ-8 as a measure of current depression in the general population. J Affect Disord.

[22] Spitzer RL, Kroenke K, Williams JBW (2006). A Brief Measure for Assessing Generalized Anxiety Disorder. Arch Intern Med.

[23] Priebe S, Huxley P, Knight S (1999). Application and Results of the Manchester Short Assessment of Quality of Life (Mansa). Int J Soc Psychiatry.

[24] Orosz G, Tóth-Király I, Bőthe B (2016). Four facets of Facebook intensity — The development of the Multidimensional Facebook Intensity Scale. Pers Individ Dif.

[25] Ellison NB, Steinfield C, Lampe C (2007). The Benefits of Facebook “Friends:” Social Capital and College Students’ Use of Online Social Network Sites. J Comput Mediat Commun.

[26] Toyama M, Godoy-Casasbuenas N, Olivar N (2022). Identifying resources used by young people to overcome mental distress in three Latin American cities: a qualitative study. BMJ Open.

[27] Gómez-Restrepo C, Diez-Canseco F, Brusco LI (2025). Mental Distress Among Youths in Low-Income Urban Areas in South America. JAMA Netw Open.

[28] Beltrána P, Trosserob A, Kardefeltb D (2019). Social media and mental health of adolescents in latin america. https://chasp.co.ke/wp-content/uploads/2019/06/Final-Report-Social-Media-and-Mental-Health-of-Adolescent-in-Latin-America.pdf.

[29] Adu A, Milmo D (2023). Rishi sunak considers curbing social media use for under-16s. https://www.theguardian.com/media/2023/dec/14/rishi-sunak-considers-curbing-social-media-use-under-16s.

[30] Alfaro A (2024). Proyecto de ley busca prohibir el uso de las redes sociales a los menores de 16 años en la florida. https://www.telemundo51.com/noticias/decision/local-decision/proyecto-de-ley-prohibe-el-uso-de-las-redes-sociales-a-los-menores-de-16-anos-en-la-florida/2502857/.

[31] Bou CP, Bou CP (2023). El parlamento europeo pide nuevas normas para combatir la adicción de los menores a las redes sociales. https://www.elperiodico.com/es/tecnologia/20231212/union-europea-normas-limitar-adiccion-redes-sociales-pantallas-moviles-jovenes-ninos-prohibicion-95735061.

