# Supplementary material for: How are different levels of social media engagement associated with mental distress and quality of life in young people living in deprived urban areas? A cross-sectional study in Bogotá, Buenos Aires and Lima
Source: BMJ Open. 2025 Sep 17;15(9):e102466. doi: 10.1136/bmjopen-2025-102466 (PMC12458863; doi:10.1136/bmjopen-2025-102466)
Supplement: online supplemental material 1 [file bmjopen-15-9-s001.docx]

**SUPPLEMENTARY MATERIAL**

**SM 1. Original Multidimensional Facebook Intensity Scale**

In the following, you are going to read items related to Facebook use. For each statement, please indicate your answer on the following scale:

1 - Strongly disagree. 2 - Disagree. 3 - Neither agree nor disagree. 4 – Agree.

5 - Strongly agree.

1. If I could visit only one site on the internet, it would be Facebook.

2. Watching Facebook posts is good for overcoming boredom.

3. I spent time on Facebook at the expense of my obligations.

4. My Facebook profile is rather detailed.

5. I feel bad if I don’t check my Facebook daily.

6. When I’m bored, I often go to Facebook.

7. I spend more time on Facebook than I would like to.

8. I like refining my Facebook profile.

9. I often search for internet connection in order to visit Facebook.

10. If I’m bored, I open Facebook.

11. It happens that I use Facebook instead of sleeping.

12. It is important for me to update my Facebook profile regularly.

13. Before going to sleep, I check Facebook once more.

**SM 2. Adapted Multidimensional Facebook Intensity Scale**

These questions refer to the reasons and frequency with which you use social media (for example, Facebook, Instagram, Twitter, Snapchat, etc., excluding WhatsApp). Please answer based on how you have used social media over the past 30 days.

1 - Strongly disagree. 2 - Disagree. 3 - Neither agree nor disagree. 4 – Agree.

5 - Strongly agree.

1. If I could visit only one type of page/site on the Internet, it would be social media.

2. I spend time on social media sites at the expense of my obligations.

3. My social media profile(s) is (are) rather detailed and contain(s) a lot of personal information.

4. I feel bad if I don't check my social media daily.

5. When I am bored, I often use social media sites.

6. I feel that I spend too much time on social media.

7. I often look for an internet connection in order to be able to log on to social media.

8. It happens that I use social media instead of sleeping.

9. Before I go to sleep, I check social media one more time.

10. I use social media to communicate with people I see in person.

11. I use social media to communicate with people I do not see in person.

12. I participate in support groups or internet forums.

13. I give up spending time with my family/friends to spend time on social media sites.

14. I feel more comfortable expressing my feelings through social media sites than in person.

15. I look for solutions to my problems on social media sites.

**Table 5. ANOVA test: PHQ-8, GAD-7, and MANSA scores, difference according to SME groups**

| **ANOVA** | | | | | | |
| --- | --- | --- | --- | --- | --- | --- |
|  | | Sum of squares | degrees of freedom | Root mean square | F score | Sig. |
| phq8_score | Between groups | 2925.854 | 3 | 975.285 | 28.232 | <0.001 |
|  | Within groups | 82736.886 | 2395 | 34.546 |  |  |
|  | Total | 85662.739 | 2398 |  |  |  |
| gad7_score | Between groups | 1496.078 | 3 | 498.693 | 21.091 | <0.001 |
|  | Within groups | 56629.204 | 2395 | 23.645 |  |  |
|  | Total | 58125.282 | 2398 |  |  |  |
| mansa_score | Between groups | 29.351 | 3 | 9.784 | 8.476 | <0.001 |
|  | Within groups | 2704.284 | 2343 | 1.154 |  |  |
|  | Total | 2733.634 | 2346 |  |  |  |

**Table 6. Tukey post-hoc test results: PHQ-8 scores comparison between SME groups**

| **Multiple comparisons - PHQ-8** | | | | | | | | |
| --- | --- | --- | --- | --- | --- | --- | --- | --- |
| Dependent variable | | (I) Social media engagement group | (J) Social media engagement group | Difference of means (I-J) | Standard error | Sig. | 95% confidence interval | |
|  |  |  |  |  |  |  | Lower limit | Upper limit |
| phq8_score | HSD Tukey | Low | Moderate | -1.392^*^ | 0.360 | 0.001 | -2.32 | -0.47 |
|  |  |  | High | -2.853^*^ | 0.382 | <0.001 | -3.83 | -1.87 |
|  |  |  | Very High | -4.845^*^ | 0.723 | <0.001 | -6.70 | -2.99 |
|  |  | Moderate | Low | 1.392^*^ | 0.360 | 0.001 | 0.47 | 2.32 |
|  |  |  | High | -1.462^*^ | 0.271 | <0.001 | -2.16 | -0.77 |
|  |  |  | Very High | -3.453^*^ | 0.671 | <0.001 | -5.18 | -1.73 |
|  |  | High | Low | 2.853^*^ | 0.382 | <0.001 | 1.87 | 3.83 |
|  |  |  | Moderate | 1.462^*^ | 0.271 | <0.001 | 0.77 | 2.16 |
|  |  |  | Very High | -1.992^*^ | 0.683 | 0.019 | -3.75 | -0.24 |
|  |  | Very High | Low | 4.845^*^ | 0.723 | <0.001 | 2.99 | 6.70 |
|  |  |  | Moderate | 3.453^*^ | 0.671 | <0.001 | 1.73 | 5.18 |
|  |  |  | High | 1.992^*^ | 0.683 | 0.019 | 0.24 | 3.75 |
| *. The difference in means is significant at the 0.05 level. | | | | | | | | |

**Table 7. Tukey post-hoc test results: GAD-7 scores comparison between SME groups**

| **Multiple comparisons GAD-7** | | | | | | | | |
| --- | --- | --- | --- | --- | --- | --- | --- | --- |
| Dependent variable | | (I) Social media engagement group | (J) Social media engagement group | Difference of means (I-J) | Standard error | Sig. | 95% confidence interval | |
|  |  |  |  |  |  |  | Lower limit | Upper limit |
| gad7_score | HSD Tukey | Low | Moderate | -0.625 | 0.298 | 0.155 | -1.39 | 0.14 |
|  |  |  | High | -1.687^*^ | 0.316 | <0.001 | -2.50 | -0.87 |
|  |  |  | Very High | -3.751^*^ | 0.598 | <0.001 | -5.29 | -2.21 |
|  |  | Moderate | Low | 0.625 | 0.298 | 0.155 | -0.14 | 1.39 |
|  |  |  | High | -1.062^*^ | 0.224 | <0.001 | -1.64 | -0.49 |
|  |  |  | Very High | -3.127^*^ | 0.555 | <0.001 | -4.55 | -1.70 |
|  |  | High | Low | 1.687^*^ | 0.316 | <0.001 | 0.87 | 2.50 |
|  |  |  | Moderate | 1.062^*^ | 0.224 | <0.001 | 0.49 | 1.64 |
|  |  |  | Very High | -2.064^*^ | 0.565 | 0.001 | -3.52 | -0.61 |
|  |  | Very High | Low | 3.751^*^ | 0.598 | <0.001 | 2.21 | 5.29 |
|  |  |  | Moderate | 3.127^*^ | 0.555 | <0.001 | 1.70 | 4.55 |
|  |  |  | High | 2.064^*^ | 0.565 | 0.001 | 0.61 | 3.52 |
| *. The difference in means is significant at the 0.05 level. | | | | | | | | |

**Table 8. Tukey post-hoc test results: MANSA scores comparison between SME groups**

| **Multiple comparisons - MANSA** | | | | | | | | |
| --- | --- | --- | --- | --- | --- | --- | --- | --- |
| Dependent variable | | (I) Social media engagement group | (J) Social media engagement group | Difference of means (I-J) | Standard error | Sig. | 95% confidence interval | |
|  |  |  |  |  |  |  | Lower limit | Upper limit |
| mansa_score | HSD Tukey | Low | Moderate | 0.05804 | 0.06699 | 0.822 | -0.1142 | 0.2303 |
|  |  |  | High | .23238^*^ | 0.07091 | 0.006 | 0.0501 | 0.4147 |
|  |  |  | Very High | .48014^*^ | 0.13326 | 0.002 | 0.1376 | 0.8227 |
|  |  | Moderate | Low | -0.05804 | 0.06699 | 0.822 | -0.2303 | 0.1142 |
|  |  |  | High | .17435^*^ | 0.05000 | 0.003 | 0.0458 | 0.3029 |
|  |  |  | Very High | .42210^*^ | 0.12341 | 0.004 | 0.1048 | 0.7394 |
|  |  | High | Low | -.23238^*^ | 0.07091 | 0.006 | -0.4147 | -0.0501 |
|  |  |  | Moderate | -.17435^*^ | 0.05000 | 0.003 | -0.3029 | -0.0458 |
|  |  |  | Very High | 0.24776 | 0.12558 | 0.199 | -0.0751 | 0.5706 |
|  |  | Very High | Low | -.48014^*^ | 0.13326 | 0.002 | -0.8227 | -0.1376 |
|  |  |  | Moderate | -.42210^*^ | 0.12341 | 0.004 | -0.7394 | -0.1048 |
|  |  |  | High | -0.24776 | 0.12558 | 0.199 | -0.5706 | 0.0751 |
| *. The difference in means is significant at the 0.05 level. | | | | | | | | |

**Table 9. Descriptive analysis of PHQ-8, GAD-7, MANSA and SME scores**

| Variables | PHQ-8 | GAD-7 | MANSA | SME |
| --- | --- | --- | --- | --- |
| Values |  |  |  |  |
| N | 2399 | 2399 | 2347 | 2399 |
| Min | 0.00 | 0.00 | 1.00 | 15.00 |
| Max | 24.00 | 21.00 | 7.00 | 75.00 |
| Mean | 10.01 | 8.51 | 4.65 | 41.81 |
| SD | 2399 | 4.92 | 1.08 | 10.51 |

**Table 10. Correlation between PHQ-8, GAD-7, MANSA, and SME as continuous variables.**

| Variables | r | 95% CI | t | df | p |
| --- | --- | --- | --- | --- | --- |
| SME - PHQ-8 | 0.19 | [ 0.16, 0.23] | 9.69 | 2397 | <.001*** |
| SME - GAD-7 | 0.16 | [ 0.12, 0.20] | 7.86 | 2397 | <.001*** |
| SME - MANSA | -0.10 | [-0.14, -0.06] | -4.92 | 2345 | .001*** |

**Table 11. Path analysis: SME as independent variable and PHQ-8, GAD-7, and MANSA scores as dependent variables.**

| IV - DV | est.std | se | z | pvalue | ci.lower | ci.upper | R2 |
| --- | --- | --- | --- | --- | --- | --- | --- |
| SME – PHQ-8 | 0.20 | 0.02 | 10.34 | < .001 | 0.16 | 0.24 | .041 |
| SME – GAD-7 | 0.17 | 0.02 | 8.45 | < .001 | 0.13 | 0.21 | .028 |
| SME – MANSA | -0.10 | 0.02 | -4.96 | < .001 | -0.14 | -0.06 | .010 |

**Table 12. Mean scores of depression, anxiety, and quality of life of the total sample and by SME group. Gender analysis**

|  | | **Mean scores** | | | | |
| --- | --- | --- | --- | --- | --- | --- |
|  |  | **Total Sample**  **N=2399*** | **Low Engagement**  **N=342** | **Moderate Engagement**  **N=1202** | **High Engagement**  **N=773** | **Very High Engagement**  **N= 82** |
| **Depression** | **Total sample** | 10.01  (SD=5.98) | 8.23  (SD=6.3) | 9.62  (SD=5.9) | 11.08  (SD=5.7) | 13.07  (SD=5.3) |
|  | **Male** | 8.30  (SD=5.7) | 7.00  (SD=6.03) | 8.05  (SD=5.76) | 9.17  (SD=5.26) | 11.20  (SD=5.91) |
|  | **Female** | 10.80  (SD=5.91) | 8.93  (SD=6.31) | 10.33  (SD=5.82) | 11.95  (SD=5.64) | 13.74  (SD=4.96) |
| **Anxiety** | **Total sample** | 8.51  (SD=4.92) | 7.53  (SD= 5.4) | 8.15  (SD=4.8) | 9.22  (SD=4.7) | 11.28  (SD=4.6) |
|  | **Male** | 7.06  (SD=4.93) | 6.30  (SD=5.47) | 6.72  (SD=4.66) | 7.75  (SD=4.45) | 9.90  (SD=4.41) |
|  | **Female** | 9.02  (SD=4.83) | 8.25  (SD=5.25) | 8.82  (SD=4.73) | 9.88  (SD=4.62) | 11.79  (SD=4.58) |
| **Quality of life** | **Total sample** | 4.65  (SD=1.08) | 4.77  (SD=1.2) | 4.71  (SD=1.0) | 4.54  (SD=1.0) | 4.29  (SD=1.3) |
|  | **Male** | 4.93  (SD=1.05) | 5.05  (SD=1.24) | 4.99  (SD=1.02) | 4.78  (SD=0.96) | 4.88  (SD=1.33) |
|  | **Female** | 4.52  (SD=1.06) | 4.61  (SD=1.20) | 4.59  (SD=1.02) | 4.43  (SD=1.03) | 4.11  (SD=1.17) |

**Table 13. Mean scores of depression, anxiety, and quality of life of the total sample and by SME group. Age group analysis**

|  | | **Mean scores** | | | | |
| --- | --- | --- | --- | --- | --- | --- |
|  |  | **Total Sample**  **N=2399*** | **Low Engagement**  **N=342** | **Moderate Engagement**  **N=1202** | **High Engagement**  **N=773** | **Very High Engagement**  **N= 82** |
| **Depression** | **Total sample** | 10.01  (SD=5.98) | 8.23  (SD=6.3) | 9.62  (SD=5.9) | 11.08  (SD=5.7) | 13.07  (SD=5.3) |
|  | **Adolescents** | 10.40  (SD=5.92) | 8.06  (SD=6.08) | 10.10  (SD=5.80) | 11.44  (SD=5.76) | 13.97  (SD=4.83) |
|  | **Young adults** | 11.86  (SD=6.00) | 8.35  (SD=6.43) | 9.25  (SD=5.99) | 10.76  (SD=5.60) | 12.44  (SD=5.52) |
| **Anxiety** | **Total sample** | 8.51  (SD=4.92) | 7.53  (SD= 5.4) | 8.15  (SD=4.8) | 9.22  (SD=4.7) | 11.28  (SD=4.6) |
|  | **Adolescents** | 8.48  (SD=4.93) | 7.15  (SD=5.18) | 8.24  (SD=4.92) | 9.10  (SD=4.67) | 11.18  (SD=4.87) |
|  | **Young adults** | 8.55  (SD=4.92) | 7.82  (SD=5.56) | 8.10  (SD=4.74) | 9.32  (SD=4.74) | 11.35  (SD=4.40) |
| **Quality of life** | **Total sample** | 4.65  (SD=1.08) | 4.77  (SD=1.2) | 4.71  (SD=1.0) | 4.54  (SD=1.0) | 4.29  (SD=1.3) |
|  | **Adolescents** | 4.76  (SD=1.10) | 5.07  (SD=1.25) | 4.83  (SD=1.03) | 4.60  (SD=1.05) | 4.17  (SD=1.45) |
|  | **Young adults** | 4.56  (SD=1.05) | 4.55  (SD=1.17) | 4.62  (SD=1.04) | 4.49  (SD=1.00) | 4.38  (SD=1.09) |

In the following, you are going to read items related to Facebook use. For each statement,

please indicate your answer on the following scale:

1 - Strongly disagree. 2 - Disagree. 3 - Neither agree nor disagree.

4 - Agree. 5 - Strongly agree.

12345

1. If I could visit only one site on the internet, it would be Facebook. OOOOO

2. Watching Facebook posts is good for overcoming boredom. OOOOO

3. I spent time on Facebook at the expense of my obligations. OOOOO

4. My Facebook profile is rather detailed. OOOOO

5. I feel bad if I don’t check my Facebook daily. OOOOO

6. When I’m bored, I often go to Facebook. OOOOO

7. I spend more time on Facebook than I would like to. OOOOO

8. I like refining my Facebook profile. OOOOO

9. I often search for internet connection in order to visit Facebook. OOOOO

10. If I’m bored, I open Facebook. OOOOO

11. It happens that I use Facebook instead of sleeping. OOOOO

12. It is important for me to update my Facebook profile regularly. OOOOO

13. Before going to sleep, I check Facebook once more. OOOOO
