# Supplementary material for: How are different levels of social media engagement associated with mental distress and quality of life in young people living in deprived urban areas? A cross-sectional study in Bogotá, Buenos Aires and Lima
Source: BMJ Open. 2025 Sep 17;15(9):e102466. doi: 10.1136/bmjopen-2025-102466 (PMC12458863; doi:10.1136/bmjopen-2025-102466)
Supplement: Supplementary file 2 [file bmjopen-2025-102466.reviewer_comments.pdf]

PEER REVIEW HISTORY

BMJ Open publishes all reviews undertaken for accepted manuscripts. Reviewers are asked to complete a checklist review form and are provided with free text boxes to elaborate on their assessment. These free text comments are reproduced below.

ARTICLE DETAILS

Title (Provisional)

How are different levels of social media engagement associated with mental distress and quality of life in young people living in deprived urban areas? A cross-sectional study in Bogotá, Buenos Aires, and Lima

Authors

Lucchetti, Santiago; Brusco, Luis Ignacio; Diez-Canseco, Francisco; Gómez-Restrepo, Carlos; Olivar, Natividad; Flores, Sumiko; Montes Guzmán, Laura; Fung, Catherine; Osorio Jaramillo, Isabela; Sanchez, Heidy; Stanislaus Sureshkumar, Diliniya; Priebe, Stefan

---

VERSION 1 - REVIEW

**Reviewer**	**1**
**Name**	**M, yogesh**
**Affiliation**	**MP Shah Government Medical College**
**Date**	**30-Apr-2025**
**COI**	**None**

Major Weaknesses

Causality and bidirectionality: While the authors acknowledge the limitation of their cross-sectional design in establishing causality, this limitation could be more thoroughly discussed. The relationship between SME and mental health may be bidirectional, with pre-existing mental health issues potentially leading to increased social media use as a coping mechanism. This deserves more substantive treatment in the discussion.

Adaptation of MFIS: The adaptation process for the Multidimensional Facebook Intensity Scale lacks sufficient detail. The authors mention adding and removing items but do not adequately address how this might affect the validity and reliability of the measure. A more rigorous description of the adaptation process and its potential impact on results is needed.

Contextual factors: Given the study's focus on deprived urban areas, more attention should be paid to contextual factors that might influence both SME and mental health in these

settings (e.g., limited alternative recreational opportunities, safety concerns, economic stressors). These factors could confound the observed associations.

COVID-19 impact: While the authors briefly mention that data collection coincided with the COVID-19 pandemic, they do not sufficiently address how pandemic-related lockdowns and social restrictions might have influenced SME patterns and mental health outcomes during this period.

Minor Issues

The abstract could more clearly state the limitations of cross-sectional design regarding causality.

Some repetition exists between the results section and tables; streamlining could improve readability.

The manuscript would benefit from more precise reporting of effect sizes to complement the statistical significance findings.

The discussion of clinical and policy implications could be strengthened with more concrete, actionable recommendations based on the findings.

Some references are relatively dated; incorporating more recent literature would strengthen the paper.

Specific Comments by Section

Introduction

The introduction provides adequate background but could benefit from a more comprehensive review of the current literature on social media use in the Global South. More explicit discussion of the unique aspects of social media use in deprived urban areas would strengthen the rationale for this specific study population.

Methods

The methods section is generally clear, but additional details would improve reproducibility:

More explanation of the MFIS adaptation process

Clearer justification for the chosen cut-points in categorizing SME levels

Information on how potential confounding variables were addressed in the analysis

Results

The results are presented clearly, with appropriate tables and figures. However:

It would be useful to include more information about potential confounding variables

Additional analysis examining possible interaction effects (e.g., between age groups or gender and SME) would enrich the findings

Discussion

The discussion interprets the findings in the context of existing literature but has several limitations:

Insufficient exploration of alternative explanations for the observed associations

Limited discussion of how the unique socioeconomic context might influence the relationship between SME and mental health

More detailed discussion of the potential mechanisms linking SME to mental health outcomes would strengthen this section

---

VERSION 1 - AUTHOR RESPONSE

Reviewer: 1<br>Dr. yogesh M, MP Shah Government Medical College		
Causality and bidirectionality: While the authors acknowledge the limitation of their cross-sectional design in establishing causality, this limitation could be more thoroughly discussed. The relationship between SME and mental health may be bidirectional, with pre-existing mental health issues potentially leading to increased social media use as a coping mechanism. This deserves more substantive treatment in the discussion.	We thank the reviewer for this suggestion. We have updated the discussion section to give this point more substantive treatment.	17-20
Adaptation of MFIS: The adaptation process for the Multidimensional Facebook Intensity Scale lacks sufficient detail. The authors mention adding and removing items but do not adequately address how this might affect the validity and reliability of the measure. A more rigorous description of the adaptation process and its potential impact on results is needed.	Thank you for noticing this relevant issue. As suggested by the reviewer and the editor, we have updated the description of the MFIS adaptation in the methods section for more clarity.	10
Contextual factors: Given the study's focus on deprived urban areas, more attention should be paid to contextual factors that might influence both SME and mental health in these settings (e.g., limited alternative recreational opportunities, safety concerns, economic stressors). These factors could confound the observed associations.	Thank you for this thoughtful comment. We have added a paragraph to the Introduction and Discussion sections highlighting how structural and environmental factors may influence both social media use and mental health outcomes. We also emphasize the importance of future research exploring how such factors shape	5/19

	the role of social media in underserved populations	
COVID-19 impact: While the authors briefly mention that data collection coincided with the COVID-19 pandemic, they do not sufficiently address how pandemic-related lockdowns and social restrictions might have influenced SME patterns and mental health outcomes during this period.	We agree that the COVID-19 pandemic may have influenced both social media engagement and mental health during the study period. In response to the reviewer's comment, we have added a paragraph in both the Discussion and Strengths and limitations sections to explicitly acknowledge this issue. However, pandemic-related restrictions in South American cities were highly heterogeneous. Our study was not designed to systematically capture these variations, and we have clarified this as a limitation.	17-18, 19-20
The abstract could more clearly state the limitations of cross-sectional design regarding causality.	As suggested, the limitations regarding causality were added to the abstract.	2
Some repetition exists between the results section and tables; streamlining could improve readability.	Thank you for this suggestion. We reviewed the results section carefully and revised the text to avoid repetition. We retained only the information that complements the tables by highlighting and contextualizing the most relevant findings.	13-15
The manuscript would benefit from more precise reporting of effect sizes to complement the statistical significance findings.	We appreciate this observation, which was also raised during the previous round of peer review. Although we appreciate the reviewer's suggestion, we categorized participants into groups to transparently illustrate differences in symptom scores, allowing for a direct interpretation of the clinical and practical significance of the findings. The categorisation reflects meaningful thresholds of scores on the questionnaire and is independent of the distribution of scores found in this study. As such, they are always transparent and replicable in further research.	-

	Since standard deviations are provided, effect sizes can be derived if needed. However, reporting effect sizes separately would not add meaningful new information, as they would still require translation into actual symptom differences, information that is already clearly presented in our findings. This approach ensures accessibility for all readers, including clinicians and policymakers, without the need for additional statistical interpretation. That said, if the editor or reviewers feel it would enhance the manuscript, we would be happy to report effect sizes in the supplementary material.	
The discussion of clinical and policy implications could be strengthened with more concrete, actionable recommendations based on the findings.	We appreciate the reviewer's suggestion and understand the interest in identifying actionable insights based on our findings. However, we prefer to take a cautious approach given the observational and cross-sectional nature of our study, which does not allow us to establish causal relationships or test interventions. In a previous round of peer review, we were advised to avoid overinterpretation. Our aim was to provide thoughtful but appropriate interpretations that could inform future research, clinical reflection, and contextual understanding, without implying causality or suggesting interventions that we have not tested.<br>However, if the editor or reviewers feel that additional clarification is needed, we are happy to include actionable recommendations with a short statement reinforcing the need for cautious interpretation.	-

Some references are relatively dated; incorporating more recent literature would strengthen the paper.	Thank you for this comment. We reviewed our reference list and confirmed that the majority of sources were published from 2020 onward. We retained a few earlier references where they remain relevant and widely used, particularly for validated instruments and key conceptual frameworks. However, if the reviewer considers that any specific references should be replaced, we would be happy to revise accordingly.	-
Introduction<br>The introduction provides adequate background but could benefit from a more comprehensive review of the current literature on social media use in the Global South. More explicit discussion of the unique aspects of social media use in deprived urban areas would strengthen the rationale for this specific study population.	Thank you for this valuable suggestion. We have revised the introduction to include a more detailed discussion of the contextual risk factors associated with mental health in deprived urban areas of the Global South. We added literature regarding structural inequalities in these contexts and further discussed potential roles of social media, strengthening the rationale for this study.	5
Methods<br>The methods section is generally clear, but additional details would improve reproducibility:<br><br>More explanation of the MFIS adaptation process	Thank you for noticing this relevant issue. As suggested by the reviewer and the editor, we have updated the description of the MFIS adaptation in the methods section for more clarity.	10
Clearer justification for the chosen cut-points in categorizing SME levels	Thank you for raising this point, we appreciate the opportunity to clarify it. The cut-points used to define SME categories were not derived from the sample distribution, but rather reflect the underlying structure of the Likert scale used in the scale. Each group corresponds to an average item response range (1–2, 2–3, 3–4, and 4–5), providing semantically meaningful and globally replicable thresholds. This approach ensures that the	11-12

	categorization is transparent, avoids data-driven or sample-specific groupings, and supports clinically relevant interpretation. This was stated in the data analysis section, but we slightly revised the text to make this rationale clearer to readers.	
Information on how potential confounding variables were addressed in the analysis	This was an exploratory analysis about the association of social media engagement with symptoms and quality of life. We did not conduct adjusted analyses or control for potential confounders. As we did not have any a priori defined hypotheses about specific confounding variables, we aimed to avoid the risk of adjusting for variables without theoretical justification, which may eliminate meaningful variance. However, we added results for subgroups by age group and gender (the two confounding factors particularly suggested by the reviewer). The scores show that the findings were relatively consistent across age groups and gender, so that those two factors have been addressed as potential confounders.	16
Results<br>The results are presented clearly, with appropriate tables and figures. However:<br><br>It would be useful to include more information about potential confounding variables	As suggested by the reviewer, we added the findings for subgroups by age group and gender. Presenting the actual score is more transparent to the reader than just including the factors in a multivariable analysis, since the reader can see the real differences as they are and the findings can also directly be used for potential systematic reviews and meta-analyses. We did not want to test the differences in these subgroups for statistical significance to avoid problems of multiple testing without additional hypothesis and because the study was not	16

	originally powered for analyses in subgroups.	
Additional analysis examining possible interaction effects (e.g., between age groups or gender and SME) would enrich the findings	As outlined in the response to the previous point, we followed the suggestion of the reviewer and provide the findings separately for the two age groups and genders. In order to avoid lengthening the manuscript too much, we summarise the results in the text and provide the actual findings in the supplementary material.	16
Discussion<br>The discussion interprets the findings in the context of existing literature but has several limitations:<br><br>Insufficient exploration of alternative explanations for the observed associations	As suggested, we have now expanded the Discussion section to present more clearly alternative explanations for the observed associations. Specifically, we discuss the potential for bidirectional causality and highlight the influence of contextual and structural factors that may affect both SME and mental health.	16-20
Limited discussion of how the unique socioeconomic context might influence the relationship between SME and mental health	Thank you for this valuable suggestion. As mentioned in response to related comments, we revised both the Introduction and the Discussion to better address how structural and socioeconomic conditions in deprived urban areas may influence the relationship between social media engagement and mental health.	5/19
More detailed discussion of the potential mechanisms linking SME to mental health outcomes would strengthen this section	We agree that understanding the mechanisms linking SME to mental health outcomes is an important area for future research. However, as our study was not designed to examine these, we believe that a more detailed discussion of potential mechanisms would involve speculation that is not supported by our data.<br>We have emphasized in the “Future research” section that identifying mechanisms is a key next step, and we refer to existing qualitative and	-

	longitudinal studies that have begun to explore this question. That said, if the editors find it necessary, we are open to carefully discuss some potential mechanisms with a short statement reinforcing the need for cautious interpretation.	
If you have selected 'Yes' above, please provide details of any competing interests: none	Thank you for pointing this out. This was an error in the submission form. None of the authors have any competing interests, and we will correct the declaration in the form to reflect this.	-
